# Development of the Dentofacial Appearance Perception Scale: Validity and reliability study

**DOI:** 10.1097/MD.0000000000041637

**Published:** 2025-03-07

**Authors:** Duygu Kürklü Arpacay, Osman Hasan Tahsin Kilic, Aysel Baser

**Affiliations:** a Department of Prosthetic Dentistry, Faculty of Dentistry Izmir Democracy University, Izmir, Turkey; b Department of Psychiatry, Faculty of Medicine, Izmir Democracy University, Buca Seyfi Demirsoy Training and Research Hospital, Izmir, Turkey; c Department of Medical Education, Faculty of Medicine, Izmir Democracy University, Izmir, Turkey.

**Keywords:** body dysmorphic disorder, dental satisfaction, distress, esthetics, perception, scale

## Abstract

Concerns about the appearance of teeth and mouth, which play a key role in communication and first impressions, are natural to some extent. However, excessive concern over these areas should raise suspicion of body dysmorphic disorder (BDD). The use of a validated and standardized screening tool in dental settings may improve the identification of BDD. This study aims to develop a validated tool to alert dentists to potential BDD cases among patients seeking cosmetic dental procedures. The research team conducted a literature review and initially created a pool of 26 items. A qualitative evaluation of the scale items was conducted by the researchers, during which each question was analyzed and discussed, resulting in the reduction of the number of items to 21. During the content validity process, 2 items were removed using the Lawshe technique, and 3 items were excluded during the exploratory factor analysis, finalizing the scale with 16 items. Participants from Izmir Democracy University’s Dental Clinic were involved in the study. Both exploratory and confirmatory factor analyses were executed to affirm the scale’s construct validity, and reliability was measured through Cronbach alpha. Statistical processing was done using Statistical Package for the Social Sciences and JASP software. After rigorous item analysis and exploratory factor analyses across 2 testing rounds, a finalized version of the questionnaire emerged. A total of 193 individuals voluntarily participated in the study, with 56.67% being female. The participants had an average age of 29.53 years (min: 18, max: 65). Only individuals presenting to the university hospital dental clinic for cosmetic reasons were included in the study. The final version of the scale, comprising 16 items across 3 factors, demonstrated adequate validity and reliability. Factor analysis accounted for 53.72% of the variance, with the factors labeled as follows: “Impairment in Functionality,” “Dental Appearance Satisfaction,” and “Preoccupation.” Confirmatory factor analysis supported the structural integrity of the scale, and overall reliability, as indicated by a Cronbach alpha of 0.90, confirmed its consistency. The Dental Appearance Perception Scale is a reliable and valid tool with the potential to screen for BDD risks and enhance treatment satisfaction in dental settings.

## 1. Introduction

Until the last 2 decades, dentistry mainly focused on problems such as tooth decay, gum disease, and structural abnormalities.^[1]^ However, with the influence of popular culture and social media, dental practices have visibly shifted toward aesthetic interventions to address appearance concerns, and cosmetic dental procedures have even become seen as necessities.^[2]^ The change in patients’ expectations has pushed dentists to focus more on cosmetic goals.^[3]^

Teeth and mouth are among the areas people are most concerned about regarding appearance.^[4]^ Concerns about these areas, which play crucial roles in communication and first impressions, are acceptable to some extent.^[5]^ However, heightened concern about their appearance should raise suspicion of body dysmorphic disorder (BDD). BDD is an under recognized and underreported psychiatric condition characterized by a preoccupation with perceived flaws that are generally unnoticed by others.^[6]^ The disorder affects approximately 2% of the population equally across sexes.^[7,8]^ Individuals with BDD may seek treatment from cosmetic and maxillofacial surgeons, orthodontists, dermatologists, etc, to address perceived defects. It is essential for doctors not to confuse BDD with body dissatisfaction, which does not cause significant distress or disrupt daily life.^[9]^ In terms of etiology, genetic and neurobiological factors play a role,^[10,11]^ along with psychological factors such as difficulty in identifying emotional facial expressions and a tendency to interpret neutral faces and situations as threatening.^[8]^ Sociocultural influences, including below-average parental care, a history of teasing, and experiences of childhood neglect or abuse, also contribute to BDD.^[12]^ In addition to their preoccupations, individuals with BDD often engage in repetitive behaviors such as mirror-checking or mental acts like comparing their appearance to others.^[6]^ BDD often has a chronic course, and spontaneous recovery is rarely observed.^[13]^ It causes high levels of psychological distress,^[14]^ leading to high suicide rates,^[15]^ psychiatric comorbidities, and impairments in psychosocial functioning.^[16]^

The prevalence of BDD in dental settings is reported to be as high as 5%, and dentists’ awareness of BDD is increasing.^[17]^ However, BDD can be difficult to detect in dental settings, and psychiatric consultation is often challenging to obtain when BDD is suspected. Patients with BDD who present to dental clinics need to be identified before aesthetic treatment because, according to previous studies, the majority of BDD patients do not benefit from aesthetic treatment, and they may even experience worsening of BDD symptoms.^[18]^ Moreover, such patients may exhibit violent or aggressive behavior towards themselves or the physician.^[19]^ There is a growing consensus that BDD should be considered a contraindication for cosmetic procedures.^[19–21]^

The use of a validated and standardized screening tool in dental settings may increase the identification of individuals who may have BDD. Unfortunately, there are no validated scales for BDD specific to dental settings. Current scales address general screening questions for BDD rather than questions tailored to dentistry.

This study aimed to develop a practical tool to alert dentists to the possibility of BDD in individuals presenting with cosmetic requests. The null hypothesis is that a valid and reliable scale can be developed to screen for BDD in dental patients.

## 2. Materials and methods

This study developed a new questionnaire called the Dentofacial Appearance Perception Scale (DAPS), aimed at alerting dentists about the likelihood of BDD, in individuals seeking cosmetic dental treatments.

### 2.1. Sample or participants

The volunteers who participated in the study consisted of patients who visited Izmir Democracy University Faculty of Dentistry, Dental Clinic between April 2023 and February 2024. Ethical approval was granted by the Izmir Democracy University Non-Invasive Clinical Research Ethics Committee (Decision no: 2023/04-04). Participants received information about the study’s nature and purpose, including details on the study’s objectives, the voluntary basis of participation, confidentiality, and data usage conditions, prior to giving written consent. This manuscript has been reviewed according to the STARD 2015 guidelines in the methods section.

In the literature, different techniques are used to determine sample size in scale development studies. To determine the suitability of the data set for factor analysis, the sample size and the strength of the relationship between items should be considered.^[22]^ According to studies on validity and reliability in the literature, the sample size of a scale should be 10 to 20 times the number of items.^[22–24]^ Since the initial version of the DAPS questionnaire consisted of 19 items, the minimum required sample size was calculated to be 190. In line with these principles, there was no need for a power analysis. A demographic questionnaire was prepared by the researchers to identify the participants. This questionnaire included questions about the age and gender of the participants. All the participants provided consent by signing the informed consent form for this study. Individuals aged 18 to 65, literate, and presenting to the university hospital dental clinic for cosmetic reasons were included in the study. Participation in the study was voluntary, and a face-to-face questionnaire was administered. Individuals seeking treatment for other dental issues, those with previous orthodontic treatment, or those diagnosed with a psychiatric illness were excluded from the study.

Within the scope of scale development studies, face and content validity are detailed in the methods section below. Construct validity and reliability steps are detailed in the findings section, and criterion validity (prediction–comparison with other scales in the literature) is detailed in the discussion section.

### 2.2. The development of DAPS

1. The first step in developing a new scale requires a literature review.^[25]^ A systematic literature review was conducted using PubMed and Google Scholar to identify existing tools related to BDD that could inform the development of a scale specifically tailored for dental patients with BDD. The search employed the keyword combination (“body dysmorphic disorder” OR “dysmorphia”) AND (“tool” OR “questionnaire” OR “instrument” OR “scale” OR “measure” OR “inventory”). Articles published in English with accessible full texts up to April 15, 2023, were included, and reference lists of included studies were also reviewed for additional relevant contributions. After the literature review, the Diagnostic and Statistical Manual of Mental Disorders, Fifth Edition (DSM-5) diagnostic criteria as well as valid and reliable scales, including the Yale–Brown Obsessive–Compulsive Scale Modified for Body Dysmorphic Disorder (BDD-YBOCS) researched by Phillips KA et al in 2014,^[26]^ the Body Dysmorphic Disorder Questionnaire-Dermatology Version (BDD-DV) scale researched by Czernecka A et al in 2023,^[27]^ and the Cosmetic Procedure Screening Questionnaire (COPS) developed by Veale D et al in 2012,^[28]^ were carefully examined for their questions.
2. Secondly, to develop the DAPS inventory, a question pool of 26 items was created by the researchers in accordance with the relevant literature.^[29]^ A qualitative evaluation of the scale items was conducted by the researchers, and the number of items was reduced to 21 by analyzing and discussing each question.
3. The third step involved studying the content and construct validity of the scale by receiving feedback from experts. At this stage, the Lawshe technique, which is the most widely used in the literature, was applied.^[30,31]^ In terms of appearance and content validity, care was taken to ensure that the evaluation team was representative of the fields of dentistry (n:3), family medicine (n:1), psychiatry (n:1), and psychology (n:4). Experts (n = 9) were asked to use the expert evaluation form to rate the comprehensibility of the items (1 = necessary/0 = not necessary/0 = needs improvement) and to suggest revisions for the items that were not understood. Content validity was determined by the content validity index (CVI) developed by Lawshe.^[30]^ To ensure content validity, Lawshe content validity ratio method was applied. In this method, the size of the evaluation panel is critically important. According to Ayre and Scally,^[31]^ a panel of 9 experts provides an adequate level of reliability for deeming an item as “essential” and minimizes the likelihood of random agreement. Therefore, a panel of 9 experts was deemed sufficient and appropriate for content and construct analysis. In the collected data, 5 items whose inclusiveness coefficient was <0.778 were revised and the evaluation form was submitted to expert opinion again.^[30,31]^ In the Lawshe second round, the items with a I-CVI higher than 0.778 were included in the measurement tool and the other 2 items (I-CVI: -0.33 and -0.56) were removed from the item pool (V20, V21). Content validity index for the items (I-CVI) and content validity indices for the scale (S-CVI) were obtained and the final form was formed according to the CVI criteria. Since the CVI for the scale was 0.96, the scale was accepted as statistically significant.^[30–33]^
4. After these studies, the item pool was organized and reduced to 19 items. Each item in this version was designed to be answered on a five-point Likert scale: “Strongly agree = 5,” “Agree = 4,” “Neutral = 3,” “Disagree = 2,” and “Strongly disagree = 1.” In the sub-dimension related to satisfaction in this draft scale, 4 out of the 5 items were reverse coded due to their meaning.
5. The pilot study was conducted to determine the validity and reliability of the final version of the developed questionnaire after receiving feedback from experts. The study was conducted in the 2022 to 2023 academic year at the Faculty of Dentistry of Izmir Democracy University on a group of nineteen fourth- and fifth-year dental students who were training in the dental clinic within patient care. These students were selected because of the ease of conducting the scale feedback session and because they were in contact with patients in the dental clinic. The questionnaire was finalized by making corrections with the samples received from the participants. With this step, appearance and content validity were completed.^[30,31,34]^

### 2.3. Construct validity analysis

Both construct validity analyses (exploratory factor analysis and confirmatory factor analysis [CFA]) should be performed when developing a new scale. The JASP statistical software package was used to conduct both exploratory factor analysis (EFA) and CFA.^[35–37]^ JASP statistics program was used to execute the EFA and CFA.

In this study, it is recommended that Barlett test should be significant^[37]^ and the Kaiser–Meyer–Olkin (KMO) index should be higher than the minimum value of 0.6.^[22,38]^ For EFA, maximum likelihood was selected on the data, factor loading was set as 0.30 and direct Promax rotation method was used. In the factor analysis, 2 items (V1 and V11) were excluded due to factor loadings below 0.30, and item V12 was removed as it formed a standalone factor without grouping with other items. Consequently, a total of 3 items were excluded from the scale at this stage.^[39]^ During the content validity process, 2 items (V20, V21) were removed using the Lawshe technique, and 3 additional items (V1, V11, V12) were excluded during the EFA, finalizing the scale with 16 items.

### 2.4. Reliability analysis

To evaluate the internal consistency of the scale, it is very important that the scale is reliable. From this point of view, reliability is the degree to which the measurement results are free from random errors and means that the measurement results are consistent and stable. There are many statistical methods for reliability calculation. Among these, the alpha coefficient method developed by Cronbach is argued to be an internal consistency estimation method that is appropriate to use when items are not scored as true–false, but as 1-3, 1-4, 1-5. The most frequently used method for reliability in literature is Cronbach alpha calculation.^[34–36,40]^

### 2.5. Statistical analysis

Statistical Package for the Social Sciences 22.0 software was used for descriptive statistics, content and content validity and exploratory factor analysis, and JASP software was used for CFA. After the factor analysis, Cronbach alpha test was conducted to measure the reliability of the scale. Descriptive statistics (means, standard deviations, and percentages) were used to describe the demographic characteristics of the sample and the distribution of responses.

The descriptive analysis was elaborated to show that the data set of the scale was reliable and valid. Sampling adequacy for factor analysis was tested using the KMO measure of sampling adequacy.^[22,41]^ The construct validity of the scale was examined using EFA, correlation analysis for the correlation between sub-dimensions, and CFA to confirm consistency with the model defined by Norris et al.^[37,38,42,43]^ Coefficients for reliability analysis were calculated according to the classical test theory (Cronbach alpha coefficients).

## 3. Results

This section presents the demographic findings of the participants, and the results of the construct validity analyses, including exploratory and confirmatory factor analyses.

### 3.1. Demographic results

Voluntary participation in the study consisted of 193 people. Thirteen of the participants did not complete the questionnaire and left it unfinished. In the scale validity and reliability study, incomplete forms (n = 13) were not included in. Of the volunteer participants (n = 180), 56.67% were female, 43.33% were male and the average age was 29.53 (min: 18, max: 74). Prior to conducting the scale validity study, Kolmogorov–Smirnov tests were utilized to assess if the data had a normal distribution. A QQ graph of the distribution was also created. Skewness values of 0.154 ± 0.181 and kurtosis values of -0.698 ± 0.36 were calculated, and it was concluded that values within + 1.5 and -1.5 were acceptable ranges for skewness or kurtosis, based on Tabachnick and Fidell guidelines.^[22]^ The findings indicated that the data had a normal distribution.

### 3.2. Results related to the validity of the scale

#### 3.2.1. Construct validity analysis

Prior to constructing validity analysis, KMO (Measure of Sampling Adequacy) value was calculated. The statistical procedures were performed and the KMO value is presented in Table 1. In the construct validity analysis of the scale, KMO value was calculated as 0.88. The Barlett test was also found to be statistically significant (*P* = .000). Therefore, since the KMO value was >0.6, it was concluded that factor analysis was feasible.^[22,37]^

**Table 1:** KMO values of the scale.

KMO and Bartlett test		
Kaiser–Meyer–Olkin measure of sampling adequacy		0.878
Bartlett test of sphericity	Approx. Chi-square	1539.568
	df	120
	Sig.	0.000

KMO = Kaiser–Meyer–Olkin.

After the statistical analysis, the factors with eigenvalues >1 below are shown in Table 2. To decide how many factors to extract, components with an eigenvalue of 1 or more were taken into consideration.^[37]^ As a result, the scale was grouped into 3 factors and these factors cover 62.21% of the test questions (the validity percentage of the scale).

**Table 2:** Explained variance and total variance of the scale.

Total variance explained							
Component	Initial Eigen values			Extraction sums of squared loadings			Rotation sums of squared loadings*
	Total	% of Variance	Cumulative %	Total	% of Variance	Cumulative %	Total
1	6.636	41.474	41.474	6.636	41.474	41.474	5.598
2	1.712	10.697	52.171	1.712	10.697	52.171	3.544
3	1.606	10.040	62.211	1.606	10.040	62.211	3.876

Extraction method: principal component analysis.

* When components are correlated, sums of squared loadings cannot be added to obtain a total variance.

**Figure 1.**
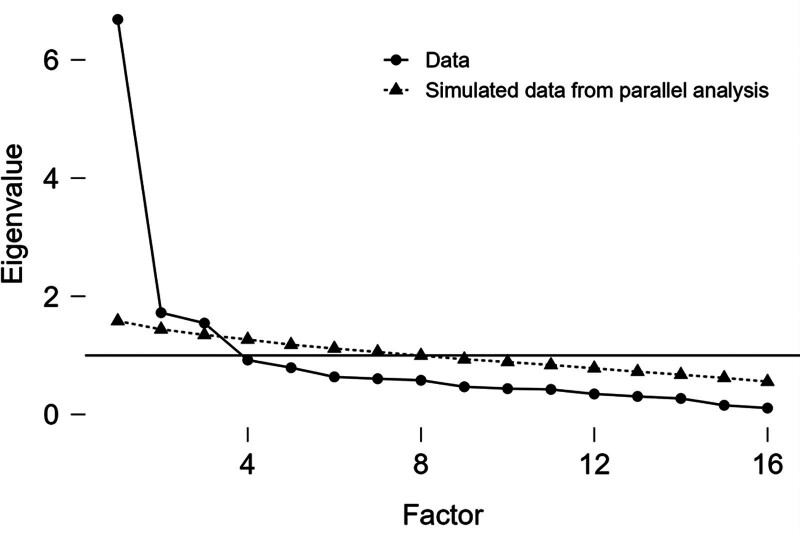
The Scree Plot graph of the scale.

In determining the factor structure of the test, the explained variance table as well as the Scree Plot graph are important visuals.^[39]^ Figure 1 shows the Scree Plot graph of the scale.

When the vertical rotations were completed according to the factor loadings, it was determined that 16 items were grouped into 3 factors. After the factor analysis of the scale, the questions in each factor were analyzed by the researcher. Factor 1 consisted of 7 items (V7, V14, V15, V16, V17, V18, and V19), factor 2 consisted of 5 items (RV2, V3, RV8, RV9, and RV10) and factor 3 consisted of 4 items (V4, V5, V6, and V13). These factors were named based on the literature review and the topics expressed by the items. Accordingly, the factors were named as follows: Factor 1: Impairment in Functioning, Factor 2: Dental Satisfaction, and Factor 3: Preoccupation. Factor loading values of the scale with DAPS questions are shown in Table 3. Many fit statistics are used in structural equation modeling^[44]^ and the most used fit indexes and observed fit values in the literature are presented in Table 4.

**Table 3:** Factor loading values of the scale.

No	Questions		Items in brief	Component Loadings				Cronbach alpha
				Fc1	Fc2	Fc3		
Q2	I am satisfied with the way I look when I smile.		Satisfaction of smile		0.725			0.74
Q8	I am satisfied with the alignment and shape of my teeth.		Satisfaction of alignment and shape		0.705			
Q10	I believe people like the look of my teeth.		Satisfaction		0.696			
Q3	I am dissatisfied with the appearance of my teeth in photographs or videos.		Dissatisfaction		0.565			
Q9	I am satisfied with the color of my teeth.		Satisfaction of color		0.554			
Q5	I am concerned about the appearance of my teeth while talking with my friends.		Preoccupation about alignment			0.906		0.82
Q4	I am concerned about the appearance of my teeth while talking with my family.		Preoccupation about color			0.773		
Q6	I am concerned about the appearance of my teeth while dating.		Preoccupation about shape			0.764		
Q13	I am concerned about the appearance of my teeth when talking to a person I don’t know very well.		Preoccupation about gum			0.458		
Q15	My thoughts about the appearance of my teeth impair my occupational functioning.		Occupational functioning	0.847				0.90
Q16	I spend a long time trying to fix the aspects of the appearance of my teeth that I don’t like. (Frequent dental checkups/visits to the dentist, looking in the mirror frequently, wearing make-up, watching dental aesthetic images on social media)		Repetitive behaviors	0.840				
Q18	My thoughts about the appearance of my teeth make me distressed.		Extensive distress	0.838				
Q14	I spend a long time comparing my teeth appearance.		Mental acts	0.808				
Q17	My thoughts about the appearance of my teeth make me depressed.		Depressed mood	0.764				
Q19	My thoughts about the appearance of my teeth impair my social functioning.		Social functioning	0.730				
Q7	When I smile, I cover my teeth with my lips or my hand.		Avoidance	0.616				
Q1		I am satisfied with the appearance of my face.					Removed	
Q11		I check the appearance of the teeth of the people around me.					Removed	
Q12		My thoughts about the appearance of my teeth have a negative impact on my social life.					Removed	

**Table 4:** Index values of the CFAFA scale.

Fit indexes	Criteria for excellent fit	Criteria for acceptable fit	Fit indexes obtained	Adaptation
χ^2^/SD	0 ≤ χ^2^/SD ≤ 2	2≤χ^2^/SD ≤ 4	2.51	Acceptable
RMSEA	0 ≤ RMSEA ≤ .05	.06 ≤ RMSEA ≤ .08	0.09	Acceptable
SRMR	0 ≤ RMSR ≤ .05	.06 ≤ RMSR ≤ .10	0.07	Acceptable
IFI	.95 ≤ IFI ≤ 1.00	.90 ≤ IFI ≤ .95	0.90	Acceptable
CFI	.95 ≤ CFI ≤ 1.00	.90 ≤ CFI ≤ .95	0.90	Acceptable
GFI	.90 ≤ GFI ≤ 1.00	.85 ≤ GFI ≤ 89	0.95	Excellent
TLI (NNFI)	.95 ≤ NNFI ≤ 1.00	.90 ≤ NNFI ≤ .94	0.88	Acceptable
NFI	95 ≤ NFI ≤ 1.00	.90 ≤ NFI ≤ .94	0.85	Acceptable

Analysis of fit indexes in structural equation modeling.

CFI = Comparative Fit Index, GFI = Goodness of Fit Index, IFI = Incremental Fit Index, NFI = Normed Fit Index, NNFI = Non-normed Fit Index, RMSEA = root mean square error of approximation, SRMR = standardized root mean square residual, Tucker–Lewis Index.

In pursuit of the most representative factor structure, a comparative evaluation of the one-factor and three-factor models was conducted. Table 4 shows that the chi-square to degrees of freedom ratio (χ^2^/SD) was 2.51, which is considered an acceptable level, slightly exceeding the excellent fit threshold. The root mean square error of approximation stood at 0.09, hovering just above the acceptable fit criterion, suggesting a marginally acceptable fit of the model to the data. The standardized root mean square residual was found to be 0.07, within the acceptable fit range and indicative of a reasonable error of approximation.

Incremental fit indices including the Incremental Fit Index, the Comparative Fit Index, and the Tucker-Lewis Index, also known as the Non-Normed Fit Index, registered at 0.90, 0.90, and 0.88 respectively; all of which align with acceptable fit according to this study’s predefined criteria. The Goodness of Fit Index excelled with a value of 0.95, indicating an excellent fit. The Normed Fit Index, with a value of 0.85, fell within the acceptable range, though on the lower end.

The analysis of fit indices suggests that while the three-factor model does not achieve excellence across all measures, it holds a generally acceptable fit to the data. Conversely, the one-factor model yielded less favorable outcomes. The root mean square error of approximation far exceeded the acceptable limit with a value of 0.139, and its confidence interval did not provide assurance of adequacy. The χ^2^/SD ratio at 4.470 signified a poor fit. While the standardized root mean square residual was somewhat close to acceptable levels, it did not meet the preferred standard. The Goodness of Fit Index, though high at 0.917, was overshadowed by the overall poor performance across other critical measures.

Considering the entirety of the evidence, the three-factor model is favored over the one-factor model. Despite some indices just bordering on acceptable levels, the three-factor structure overall provides a more adequate fit for the data, as opposed to the one-factor model which demonstrates considerable deficiencies in key areas. The structural validity of the 16-item, three-factor scale is thus upheld, and the relationships within the model are substantiated, reinforcing the conceptual framework of the scale.

**Figure 2.**
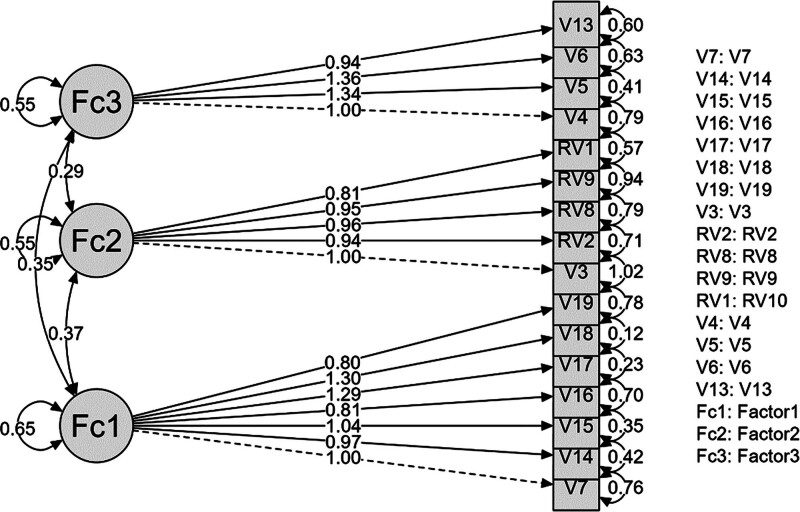
CFA model – path diagram. CFA = confirmatory factor analysis.

This structural model comprises 16 observed variables (V4, V5, V6, V7, V13, V14, V15, V16, V17, V18, V19, V3, RV10, RV2, RV8, and RV9) that are explained by 3 latent factors (Factor 1, Factor 2, and Factor 3). Each latent factor in the model is significantly related to the observed variables loaded onto it, indicated by the factor loadings, which are coefficients expressing the relationship between the observed variables and the factors. For instance, Factor 1 (Fc1) shows the strongest loading with the V4 variable (0.79), while the weakest loading is with the V18 variable (0.12). Inter-factor correlations are also included in the model; the high correlation between Factor 2 (Fc2) and Factor 3 (Fc3) (0.55) suggests that these 2 factors explain a significant portion of the variance shared with the observed variables. Overall, this model appears to be conceptually consistent with our hypotheses and exhibits a sufficient fit with the observed data (Fig. 2).

#### 3.2.2. Reliability study

In this scale development study, Cronbach alpha value was calculated to determine the reliability of the scores obtained by the participants. The values obtained are given in Table 5. When Table 5 is examined, it is seen that the reliability coefficient of the scale is 0.90. Therefore, it is concluded that the scale is reliable. In the light of all these considerations, scale scoring patients’ dentofacial perceptions was developed with validity and reliability studies. Participants responded to each item on a five-point Likert scale as “strongly agree = 5,” “agree = 4,” “undecided = 3,” “disagree = 2,” and “strongly disagree = 1.” The minimum score of the scale is 16 and the maximum score is 80. The higher the score, the worse the level of dentofacial perception. Items 2, 8, 9, and 10 in the scale should be calculated by reverse coding due to their meaning. The 48 points or three averages obtained from the scale indicate that the participants’ dentofacial perceptions are at an average level. As the scores obtained from the scale increase above the average value, it shows that the participants’ perceptions of dentofacial appearance are negative.

**Table 5:** Reliability coefficient value.

Frequentist scale reliability statistics	
Estimate	Cronbach α
Point estimate	0.90
95% CI lower bound	0.88
95% CI upper bound	0.92

*Note*: Of the observations, pairwise complete cases were used.

## 4. Discussion

The aim of this study is to develop a valid and reliable scale to screen for BDD among individuals seeking cosmetic dental treatments. The frequency of people with BDD visiting dental clinics for cosmetic reasons has been increasing, and identification of BDD is critical before cosmetic procedures.^[45]^ Therefore, having a scale that accurately screens for BDD in a dental setting would be valuable for clinical practice and patient management.

Among the BDD diagnostic/screening tools, BDD examination (BDDE) requires a lengthy face-to-face interview with a field-trained specialist, has a complex scoring system, and is not recommended for use in cosmetic settings.^[18,46,47]^ The self-administered version of BDDE, BDD examination-self-report, has been used as a diagnostic tool in cosmetic surgery patients.^[48]^ Although it has acceptable internal consistency and test–retest reliability, it has not been validated in dental settings and has not been updated according to DSM-5 changes.^[6,19]^

Body dysmorphic disorder questionnaire (BDDQ) is a brief self-assessment tool.^[49]^ It assesses BDD according to DSM-5 criteria and has validated versions for dermatology (BDDQ-DV)^[27,50]^ and plastic surgery (BDDQ-AS).^[51,52]^ Therefore, it is recommended for screening for BDD in plastic and reconstructive surgery, otolaryngology, and head and neck surgery,^[53]^ but it has not been validated in dental settings. The body image disturbance questionnaire tool is a slightly altered version of the BDDQ and BDDQ-DV scales. The scale has been validated in Turkish, but it is based on the DSM-4 criteria, which do not include the repetitive behaviors added in the last version.^[54]^ Cosmetic procedure screening has high sensitivity, acceptable internal consistency, and test–retest reliability.^[28]^ An unvalidated version of COPS was used to determine BDD prevalence in dental settings.^[55]^ The BDD-screening test was created using 2 scales: 9 questions from BDDQ-DV and 11 from BDDE, totaling 20 questions.^[56]^ Like COPS, it has also been used to determine BDD prevalence in dental settings, but it lacks validation in these settings and none of its questions are specific to dental settings. Additionally, the purpose of studies applied COPS and BDD-screening test was to determine BDD prevalence in dental settings rather than developing a screening tool specific to these settings. The Yale–Brown Obsessive–Compulsive Scale Modified for Body Dysmorphic Disorder is the most widely used measure of BDD severity in psychiatric studies.^[26]^ The scale demonstrates strong reliability, validity, and sensitivity. Although there is a Turkish adaptation,^[57]^ it is not self-reported so it is time consuming, not specific to dental settings, and has not been validated in dental settings. Another scale used in previous dental studies, the Dysmorphic Concern Questionnaire, does not precisely diagnose or screen for BDD but instead evaluates individuals’ concerns about their physical appearance. Since Dysmorphic Concern Questionnaire is not specific to BDD and requires more detailed psychometric analyses, it is not recommended for healthcare providers to use it alone for BDD screening.^[53]^

The DAPS is validated within dental settings and differs from existing scales by focusing on dental-specific preoccupations, such as teeth alignment, color, and overall smile aesthetics. It is based on DSM-5 criteria for BDD and includes an item addressing social media use, reflecting a modern version of repetitive behavior. The DAPS do not only screen for BDD but also assess individuals’ satisfaction with their dental appearance. These features make the DAPS a unique and robust scale specifically designed for dental practice.

The preliminary version of the scale consisted of 19 questions based on BDD diagnostic criteria and other BDD scales. According to expert opinions and data collected through survey questions, the number of questions was reduced to 16. The DAPS has 3 factors: impairment in functionality, dental appearance satisfaction, preoccupation. For the final version of the scale: the Cronbach alpha value for the general scale reliability was 0.90, the Cronbach alpha value for the impairment in functionality sub-factor was 0.90, the dental appearance satisfaction was 0.74, the preoccupation was 0,82 and the highest score to be obtained from the scale is 80 and the lowest score is 16. No cutoff point has been determined for the scale. A high overall score on the scale indicates the risk of BDD. The validity of the three-factor DAPS developed was proved by the CFA. When the goodness of fit criteria in the CFA were considered, it was determined that the designed model was of an acceptable structure. The CFA revealed that DAPS can be detected with these 6 diagnostic criteria: satisfaction from dental appearance, preoccupation about dental appearance, repetitive behaviors and mental acts responding preoccupation, and excessive distress/loss of functioning.

The first factor, “Impairment in Functionality” corresponds to Criteria B and C of the DSM-5.^[6]^ It assesses repetitive behaviors or mental acts, such as checking, grooming, reassurance seeking, comparing, that occur in response to appearance concerns (Criterion B) and excessive distress or impairment in functioning that occur because of preoccupation (Criterion C). Additionally, this factor includes an item that questions social media use, which can be evaluated within the framework of repetitive behavior. This item ensures the scale’s relevance to today’s digital age and provides insight into how online behaviors may contribute to BDD symptoms.

The second factor, termed “Dental Appearance Satisfaction,” measures the level of satisfaction with dental appearance. High scores on this subscale suggest significant dissatisfaction. High scores may indicate a warning sign for BDD. However, this factor was not adapted from any diagnostic criteria from DSM-5 for BDD. This subscale alone may be a valuable tool in assessing patients’ satisfaction with dental appearance and, when applied before and after cosmetic procedures, may indirectly assess the success of aesthetic interventions in individuals without BDD. However, scores are unlikely to change significantly in individuals with BDD after cosmetic interventions because after such procedures their perceptions usually remain consistent, or their preoccupation shifts to another aspect of their appearance.^[58]^

The third factor, “Preoccupation,” includes items that align with DSM-5’s Criterion A for BDD. This criterion focuses on the individual’s preoccupation with perceived defects or flaws in physical appearance, which are not observable.^[6]^ This alignment ensures that the scale directly addresses the core symptomatology of BDD, providing a critical measure for identifying potential cases in clinical settings.

This study introduces the DAPS, a validated and reliable screening tool specifically developed to identify signs of BDD within dental settings. The DAPS represents a unique advancement by bridging the gap between aesthetic dentistry and mental health, addressing patients’ aesthetic concerns while providing clinicians with a structured means to detect early indicators of BDD. This integration of mental health awareness within dental practice is particularly valuable in cosmetic dentistry, where managing patient expectations and screening for underlying psychological conditions can significantly influence treatment outcomes and patient satisfaction.

One of DAPS’s primary strengths is its ability to aid dental practitioners in identifying undiagnosed BDD cases. BDD patients frequently seek aesthetic treatments instead of psychiatric support, leading to delays in diagnosis and prolonging distress. With early identification facilitated by the DAPS, dentists can appropriately refer high-risk individuals for psychological evaluation, improving access to timely care and supporting a holistic approach to patient health. Given the high prevalence of suicidal ideation and attempts among BDD patients, this scale also offers clinicians an important tool for identifying individuals at increased risk, enabling precautionary measures that could ultimately be lifesaving.

Additionally, by reducing the likelihood of posttreatment dissatisfaction and potential complications, the DAPS enhances overall patient satisfaction and promotes more ethical, informed decision-making regarding cosmetic procedures. In this way, the DAPS does not merely function as a screening tool but serves as a critical step in preventing unnecessary or harmful interventions. The DAPS thus exemplifies a dual-purpose utility: it supports the mental health needs of patients while also fostering improved clinical outcomes in aesthetic dentistry. This dual approach underscores the importance of integrating mental health considerations into dental practice, particularly in an era where the pursuit of aesthetic enhancements is increasingly prevalent. Furthermore, the development of the DAPS addresses a gap in the literature and clinical practice by focusing on an under examined intersection of aesthetic concerns and mental health. This tool contributes to a more holistic model of dental care, aligning with the growing recognition of mental health’s impact on physical health and patient satisfaction. By offering a reliable, validated means to screen for BDD, the DAPS equips dental practitioners with a tool that can elevate standards of care in dental aesthetics, reduce potential harm, and support patients in achieving safer, more realistic aesthetic outcomes.

Perceptions of dental aesthetics vary considerably based on individual, social, and cultural factors.^[59]^ Studies indicate that aspects such as malocclusion, facial asymmetry, and gingival display contribute to differing aesthetic perceptions, and these can vary significantly across cultures. While BDD is reported globally, variations in aesthetic ideals highlight the need for cross-cultural adaptations of the DAPS to ensure its relevance in diverse populations. Furthermore, the study’s reliance on a sample from a single dental clinic may limit the generalizability of its findings. Future research across varied cultural and clinical settings would strengthen the reliability and applicability of DAPS, enhancing its utility for diverse patient populations.

Before the scale was developed, similar scales were reviewed first. However, there was no appropriate scale for the Turkish context regarding the participants’ perception levels on dentofacial appearance perception. For this reason, the reliability and validity results of the DAPS could not be compared with Turkish similar scales and could not be discussed sufficiently. Another limitation of the study is the use of a single sample. Since there was no problem in structural model fit and no item was removed, no resampling was performed. The validity of the DAPS will increase with future studies using separate samples from the same population.

## 5. Conclusion

In conclusion, the DAPS presents a robust tool for assessing BDD risk within dental contexts. Its three-factor structure aligns closely with DSM-5 criteria, ensuring clinical relevance. The scale’s high reliability and acceptable validity make it a valuable resource for enhancing patient care in cosmetic dentistry. While further validation studies are needed, the DAPS fills a critical gap in BDD screening within dental settings, offering potential for early detection and improved treatment outcomes.

## Author contributions

**Conceptualization:** Duygu Kürklü Arpaçay, Osman Hasan Tahsin Kiliç, Aysel Baser.

**Data curation:** Aysel Baser.

**Formal analysis:** Aysel Baser.

**Methodology:** Duygu Kürklü Arpaçay.

**Project administration:** Osman Hasan Tahsin Kiliç.

**Supervision:** Osman Hasan Tahsin Kiliç.

**Validation:** Aysel Baser.

**Writing – original draft:** Duygu Kürklü Arpaçay, Osman Hasan Tahsin Kiliç.

**Writing – review & editing:** Duygu Kürklü Arpaçay, Osman Hasan Tahsin Kiliç.
